# Case of Difficult Labour

**Published:** 1839-12-28

**Authors:** Orlando Fairfax

**Affiliations:** Alexandria, (D. C.)


### CASE OF DIFFICULT LABOUR.

BY ORLANDO FAIRFAX, M.D.

To the Editors of the Medical Examiner.

*Gentlemen,*—I send you the following case, thinking that at least one of its features, the occurrence of respiration twenty-four hours before birth, may make it interesting to the readers of the Medical Examiner.

Very respectfully, yours,

O. Fairfax.

On December 13th, at 11 o'clock, P.M., I was called to Mrs. M., in labour with her second child. She had been having regular pains for about three hours, and the membranes had just broken. I found the mouth and nose presenting in the os uteri, which was rigid, and dilated to an extent not greater than the size of a half dollar, the face being situated transversely in regard to the pelvis, and so high up that I could not reach it without introducing more than one finger. At 1 o'clock, A.M., in the absence of pain, having my hand in the vagina, with the points of my fingers on different parts of the face around the circumference of the os uteri, which was now dilated to twice the size of a dollar, I was surprised by hearing distinctly a sob, and then the sound of pretty regular respiration; and, shifting a finger to the mouth, I could plainly perceive a motion of the lips, synchronous with each respiratory sound. The air must have been admitted through the sulcus formed in the palm of my hand, by the approximation of the thumb to the little finger. At this time the head was engaged in the superior strait, the face being about midway in the pelvis. I did not attempt to reproduce the phenomenon, from fear of impairing the fœtal circulation. The labour progressed but slowly through the day, the os uteri being still rigid in the early part of the afternoon, though the pains had continued to be strong, and the patient had been freely bled from the arm. At 8 o'clock, P. M., the head having for several hours ceased to descend, and finding the os uteri well relaxed, and the chin inclining toward the sacrum, I introduced my hand, raised the head slightly, and rotated the chin to the pubis; the next pain caused the head to descend, bringing the base of the lower jaw into the arch of the pubis, but inclined somewhat to the right descending ramus, and the point of the chin, the mouth and nose to a situation just within the vulva. I then thought all would be well, but how great was my disappointment, when, after the next pain, I found that the chin had turned to the right sacro-ischiatic ligament, from which I had but just removed it. I repeated my manœuvre, and held the chin during four or five strong pains, without even the slightest advance occurring; in every pain the chin constantly and strongly tending to return to its former unfavourable position. I then, with the advice of my friend, Dr. F. S. Murphy, whom I had called in, determined to use the forceps. I accordingly drew off the water from the bladder, and attempted to introduce the instruments; but was foiled by the almost incessant contraction of the womb, and bearing-down efforts of the woman, excited by the introduction of the first blade. We then determined to administer to her a hundred drops of laudanum, and to leave her undisturbed for an hour. At the expiration of which time I bled her largely, brought the chin again to the pubis, introduced the instruments with comparative ease and extracted the head. The delivery of the head was accomplished at one o'clock, A. M. of the 15th. inst., twenty-four hours after my having observed the respiration. The child weighed eight pounds and a half, the head being relatively large. The mother and child are doing well.

*Alexandria*, (D. C.) December 17th, 1839.

